# Editorial: New insights into the molecular basis of long-term plasticity underlying addiction and addictive behaviors

**DOI:** 10.3389/fnmol.2023.1226345

**Published:** 2023-06-12

**Authors:** Juan Marín-Lahoz

**Affiliations:** ^1^Neurology Department, Hospital Universitario Miguel Servet, Zaragoza, Spain; ^2^Grupo de Investigación en Neurociencias, IIS Aragón, Zaragoza, Spain; ^3^Center for Biomedical Research on Neurodegenerative Diseases (CIBERNED), Madrid, Spain

**Keywords:** addiction, synaptic plasticity, behavioral addictions, animal models, molecular pathways

As editor of this Research Topic on neurobiology of addictions I am glad to present the interesting manuscripts that are finally included. I feel really grateful for the reviewers who have done a thoughtful job and for Frontiers that facilitates the process of producing a Research Topic enormously. But the ones that more strongly deserve my gratitude are the authors who not only shared their work with us but also trusted us to take care of it, review it and suggest changes.

This Research Topic contains 10 manuscripts. There is no reason to believe that this sample is completely representative of the current scientific production on addiction neurobiology. Nevertheless, it is tempting to check how the focus of the manuscripts is distributed, and also serves to introduce them.

From the point of view of the **mechanisms**, the sample is varied. As expected, much of the research evaluates synaptic plasticity and neurotransmitter pathways. Nevertheless the studied pathways are really diverse and barely overlap. Dopamine is obviously there, but only one manuscript really focuses on **dopaminergic pathways** (Bimpisidis et al.). Other neurotransmitter pathways that are explored in this topic are **glutamate** (Giangrasso et al.), and **cannabinoid** for drugs other than cannabinoids (Lucente et al.), while another evaluates the interaction of two pathways: **sigma and adenosine** (Borroto-Escuela et al.).

Nevertheless some manuscripts do not focus on neurotransmitters. Other manuscripts focus on intracellular pathways: **rho GTPases** (Ru et al.) and **Glycogen synthase kinase-3β** (Yan et al.). Some focus on the relationship between neurons from a point of view other than neurotransmitters, this is the case of the works focusing on **neuronal excitability** (Gimenez-Gomez et al.; Domi et al.) and mRNA expression of **cell adhesion molecules** (Munoz et al.). Last but not the least, one manuscript is not focused on the mechanisms but is mainly **behavioral**, focusing on the preferences to different drug concentrations (Schneider et al.).

Regarding the **species**, the two reviews include several species, one uses **zebrafish larvae** (Schneider et al.), two use **mice** (Bimpisidis et al.; Yan et al.) and all the rest use **rats**. This distribution of preclinical models seems to match the global trend but it also shows how when focusing on the internal mechanisms most of what we know about **humans** is extrapolated from animal models. There are obvious reasons why it is easier (and more efficient) to research neurotransmitter pathways, RNA expression or synapse structure in these animal models than in humans. But for the most part, the real target is to understand human addiction and the tools to investigate these mechanisms in human subjects are increasingly available: nuclear imaging, MRI connectivity, microRNA expression, and exosomes might be considered among the options.

Addiction is about **drugs**. Or is it not? Regarding the object of addiction, all of the manuscripts focus on substances. More concretely, each of them focus on a substance except for one which reviews a target for **multiple substances** (Ru et al.). Interestingly all of the manuscript but one focus on substances usually classified as drugs: **ethanol** (Gimenez-Gomez et al.), **nicotine** (Lucente et al.; Domi et al.; Schneider et al.), **methamphetamine** (Munoz et al.; Yan et al.), and **cocaine** (Borroto-Escuela et al.). The one not focused on a drug, focuses on **sucrose** (Bimpisidis et al.). Sucrose is a common sugar in modern diets and is usually referred to as a nutrient and not a drug. Nevertheless, most of the population in the history of humankind had little or no access to sucrose in a purified form and most natural foods contain no sucrose or little sucrose. Sucrose has no direct effect on reward circuits, contrary to substances considered drugs. Accordingly, the work of Bimpisidis et al. fosters the understanding of reward processing in the absence of drugs, a paramount topic to understand **behavioral addictions**, notwithstanding its usefulness to understand the addictions in which sweets are involved. As a researcher myself, I have focused my previous work in addictions not directed toward drugs but toward behaviors. Some of their mechanisms may be shared with substance overuse disorder but the knowledge on the mechanisms of behavioral addictions lags behind that of the mechanisms of drug addiction. The burden of this kind of behavior is also high and possibly growing. But the knowledge of the knowledge of its intimate mechanisms lags behind that of drug addiction.

Yet, this Research Topic shows a great predominance of drug studies over studies targeting other objects of addiction. This predominance has occurred despite an inclusive Research Topic title: New Insights Into the Molecular Basis of Long-Term Plasticity Underlying Addiction and *Addictive Behaviors*. It has also occurred despite my efforts to engage researchers focused on behavioral addictions (due to my aforementioned interest). As I stated above, this Research Topic, does not have to be a representative sample. And maybe the current research in the neurobiology of behavioral addictions is blooming somewhere else. But maybe the researchers focused on behavioral addictions are not the ones focused on neurobiology and vice versa.

I hope this trend changes in the near future with more efforts to understand the neurobiology of behavioral addictions, and no decrease in the efforts to understand drug effects. In the meantime, I hope you find this Research Topic useful and enjoy the reading.

## Author contributions

The author confirms being the sole contributor of this work and has approved it for publication.

